# Vessel Pulse Amplitude Mapping in Eyes With Central and Hemi Retinal Venous Occlusion

**DOI:** 10.1167/tvst.12.1.26

**Published:** 2023-01-24

**Authors:** Ying J. Khoo, Dao-Yi Yu, Anmar Abdul-Rahman, Chandra Balaratnasingam, Fred K. Chen, Ian L. McAllister, William H. Morgan

**Affiliations:** 1 Centre for Ophthalmology and Visual Science, The University of Western Australia, Perth, Western Australia, Australia; 2 Lions Eye Institute, Nedlands, Western Australia, Australia; 3 Department of Ophthalmology, Counties Manukau District Health Board, Auckland, New Zealand; 4 Ophthalmology Department, Royal Perth Hospital, Perth, Western Australia, Australia; 5 Ophthalmology, Department of Surgery, The University of Melbourne, East Melbourne, Victoria, Australia; 6 Centre for Eye Research Australia, The Royal Victorian Eye and Ear Hospital, East Melbourne, Victoria, Australia

**Keywords:** central retinal vein occlusion (CRVO), hemiretinal vein occlusion (HVO), venous pulsation, arterial pulsation, photoplethysmography (PPG)

## Abstract

**Purpose:**

The purpose of this study was to describe vessel pulse amplitude characteristics in eyes with central retinal vein occlusion (CRVO), hemiretinal vein occlusion (HVO), normal eyes (N1 N1), and the unaffected contralateral eyes of CRVO and HVO eyes (N1 CRVO and N1 HVO), as well as the unaffected hemivessels of HVO eyes (N2 HVO).

**Methods:**

Ophthalmodynamometry estimates of blood column pulse amplitudes with modified photoplethysmography were timed against cardiac cycles. Harmonic analysis was performed on the vessel reflectance within 0.25 to 1 mm from the disc center to construct pulse amplitude maps. Linear mixed modeling was used to examine variable effects upon the log harmonic pulse amplitude.

**Results:**

One hundred seven eyes were examined. Normal eyes had the highest mean venous pulse amplitude (2.08 ± 0.48 log u). CRVO had the lowest (0.99 ± 0.45 log u, *P* < 0.0001), followed by HVO (1.23 ± 0.46 log u, *P* = 0.0002) and N2 HVO (1.30 ± 0.59 log u, *P* = 0.0005). N1 CRVO (1.76 ± 0.34 log u, *P* = 0.52) and N1 HVO (1.33 ± 0.37 log u, *P* = 0.0101) had no significantly different mean amplitudes compared to N1 N1. Arterial amplitudes were lower than venous (*P* < 0.01) and reduced with venous occlusion (*P* < 0.01). Pulse amplitude versus amplitude over distance decreased along the N1 N1 vessels, with increasing slopes observed with CRVO (*P* < 0.01).

**Conclusions:**

Pulse amplitude reduction and attenuation characteristics of arteries and veins in venous occlusion can be measured and are consistent with reduced vessel wall compliance and pulse wave transmission.

**Translational Relevance:**

Retinal vascular pulse amplitudes can be measured, revealing occlusion induced changes, suggesting a role in evaluating the severity and progression of venous occlusion.

## Introduction

Retinal vein occlusion is the second most common retinal vascular disorder following diabetic retinopathy^1^ and a significant cause of vision loss.^2^ Retinal vein occlusion prevalence was 5.2 per 1000. It was estimated that 16 million people might have had this condition in 2008 in Europe, Asia, Australia, and the United States.^3^ Branch retinal vein occlusion (BRVO) and central forms of venous occlusion (central and hemiretinal vein occlusion) may differ in pathogenesis and risk factors.^4^^–^^8^ Our study focuses on the central forms of retinal venous occlusion, with glaucoma and ocular hypertension as significant risk factors.^4^^,^^8^^,^^9^ These risk factors have also been associated with elevated venous pulsation pressure (VPP),^10^^–^^15^ possibly due to induced elevated pressure gradients across the lamina cribrosa and corresponding central retinal vein.^16^ Due to the high prevalence of glaucoma in central retinal vein occlusion (CRVO) and hemiretinal vein occlusion (HVO) eyes suffering from glaucoma^17^^–^^20^ and its effect on venous pulsation, we included subjects with glaucoma in our study.

The VPP is the minimum (threshold) intraocular pressure (IOP) at which retinal vein pulsations can be observed.^21^ Venous pulsation pressure is known to be elevated in CRVO,^22^ and has been used to prognosticate CRVO as well as monitor the degree of occlusion and response to therapy.^23^ However, several methodological problems with VPP exist. The VPP measurement requires observer detection of minimally visible pulsation under a range of optical, illumination, and observer conditions, which limits reproducibility. In addition, the measurement is a single threshold value per vessel, usually per eye and does not provide an estimate of pulsation distribution along the vessel.^24^ It is known that venous pulsation pressure is zero (or notionally negative) in 98% of normal subjects and 54% of patients with glaucoma.^15^ The mean VPP is reported to be 4.2 Meditron units above baseline IOP in normal subjects compared to 58 Meditron units above baseline IOP in patients with CRVO, where a Meditron unit is 0.89 mm Hg.^25^^–^^27^

Photoplethysmography (PPG) objectively measures pulse amplitudes at points along the vessels and could reduce variability by removing the subjective observer component. The PPG determined pulse amplitude measurements are strongly correlated with venous pulse detection by observers and have great sensitivity (area under receiver operating characteristic curve of 0.936).^28^ The PPG has been used to estimate intracranial pressure with acceptable accuracy (mean absolute difference between lumbar puncture/ continuous measurement from external ventricular drain and PPG of 2.4 mm Hg [SD = 1.7]).^29^

We aim to objectively measure retinal vascular pulse amplitudes and their distributions in various forms of venous occlusion using PPG. As the occlusion occurs centrally, it potentially impedes pulse wave transmission from the cerebrospinal fluid compartment into the eye.^21^ If significant vessel amplitude changes are found, then such measurements may be useful clinically in assessing, prognosticating, and monitoring retinal venous occlusions.

## Methods

We recruited 70 participants from Lions Eyes Institute.

Inclusion criteria:

1. Patients who were diagnosed with central retinal vein occlusion and hemivein occlusion.
2. Patients and volunteers who had healthy eyes.

Exclusion criteria:

1. Presence of diabetic retinopathy or ischemic optic neuropathy, based on clinical evaluation, slit lamp examination, and optical coherence tomography (OCT).
2. Participants who were unable to maintain fixation during slit lamp examination.
3. Participants with significant corneal haze or media opacity.

This research was conducted in compliance with ethics approved by the University of Western Australia Human Ethics Committee, adhering to the tenets of the Declaration of Helsinki. Informed consent was obtained from all participants after explaining the nature and possible consequences of the study. All participants were reviewed using a slit lamp and indirect ophthalmoscopy, during which the diagnosis of venous occlusion was made. All venous occlusive diagnoses were confirmed by retinal specialists at our institution. All participants had automated visual field (24‐2 Humphrey Field Analyzer), OCT (Heidelberg Spectralis OCT; Heidelberg Engineering, Inc., Heidelberg, Germany) and IOP (Goldmann applanation tonometry) measured. Diagnosis of primary open angle glaucoma (POAG) was made according to matching visual field (VF), disc excavation, neuroretinal rim thinning, and nerve fiber layer loss. The participants’ pupils were dilated with 2 drops of 1% Tropicamide 10 minutes apart and rested for half an hour before undergoing ophthalmodynamometry.

### Ophthalmodynamometry Technique

We examined participants on a slit lamp (Carl Zeiss, Oberkochen, Germany). Ophthalmodynamometry (Meditron GmbH, Poststrasse, Völklingen, Germany) was used to measure the force (ophthalmodynamometric force [ODF; Meditron Unit {mu}]) applied to the eye. The Meditron Ophthalmodynamometry is constructed of a conventional Goldmann three-mirror contact lens that enables direct visualization of the posterior pole, and a force transducer ring attachment made of flexible copper-beryllium trabeculae.^2^ Force applied to the eye was measured in Meditron Unit (mu), where 1 mu = 3.33 g of force. We calculated the induced IOP using our previously described formula: Induced IOP = 0.89 × ODF + baseline IOP.^25^

**Figure 1.**
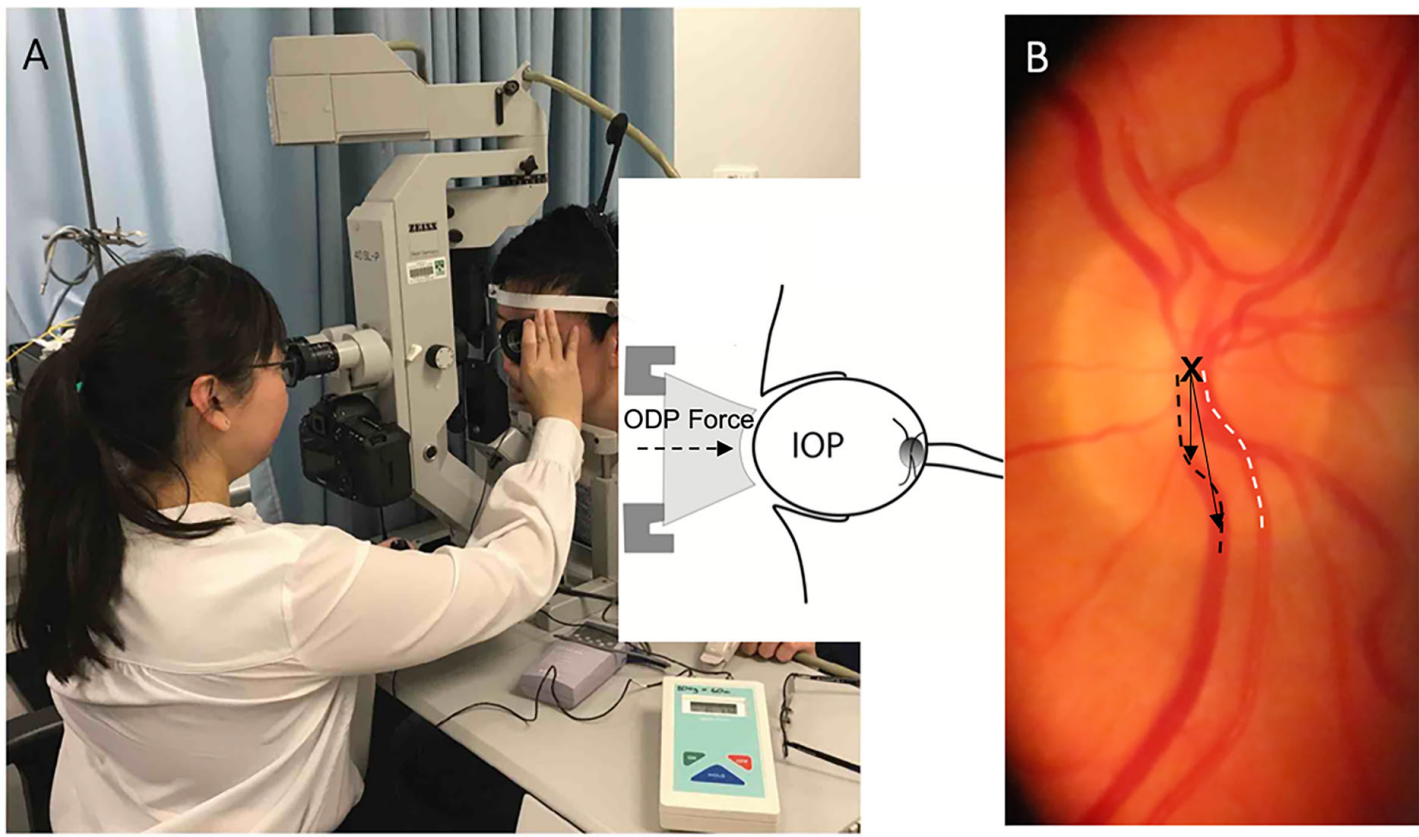
(**A**) Shows ophthalmodynamometry with simultaneous video recording of the disc and peripapillary retina on a slit lamp for photoplethysmography. (**B**) Shows a video frame captured with the *dashed lines* as sites for vessel measurements along the vein (*black*) and artery (*white*). The X is the disc center serving as a coordinate center to determine the distance of each locus along the vessels for analysis of vessel amplitude. The two *black arrows* show examples of how distance from the disc center to each locus is measured.

**Figure 2.**
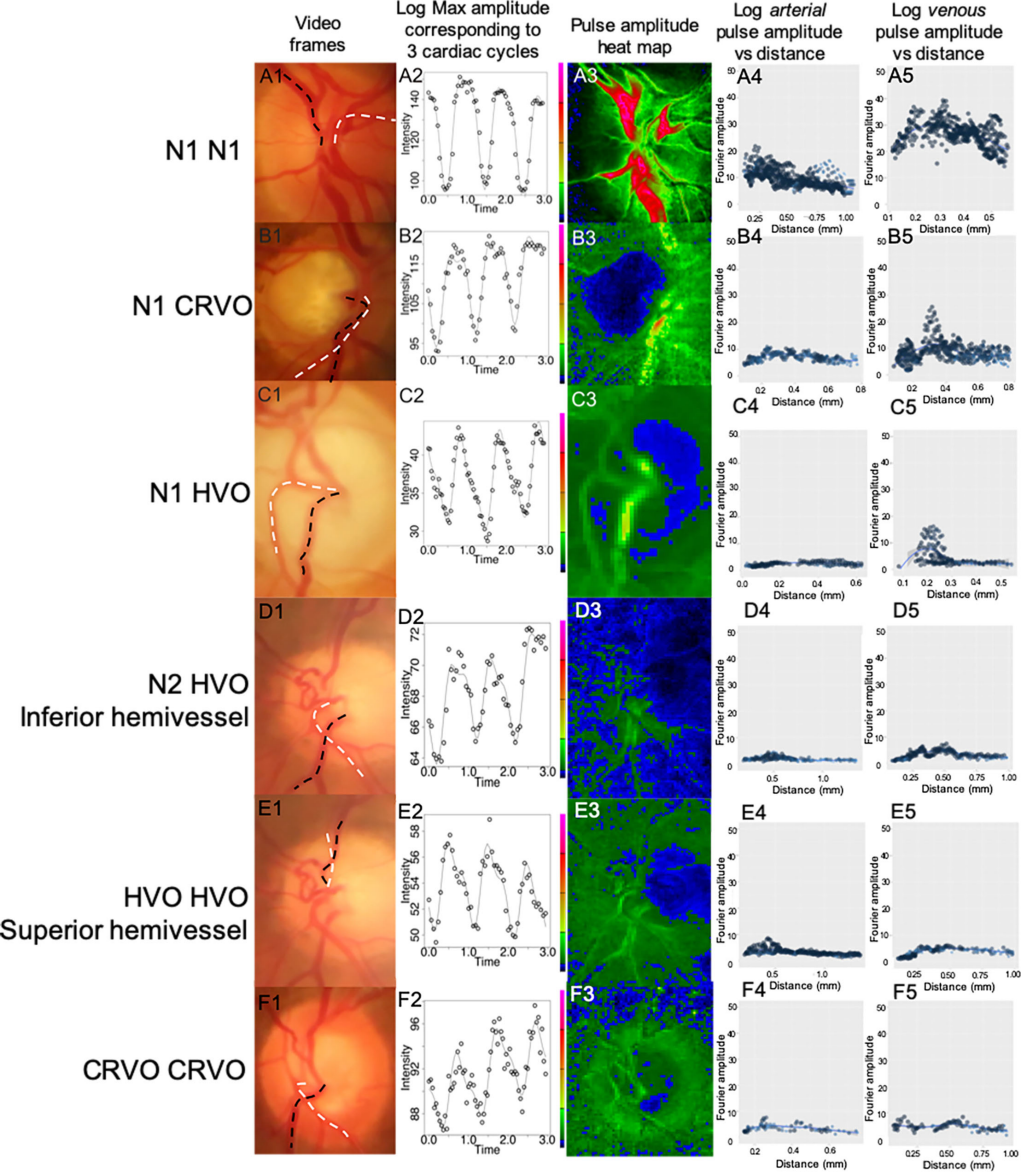
Examples of analysis sequence on each diagnosis illustrated in five columns.: A1 to F1 are video frames captured with dashed lines as sites for vessel measurements along the veins (*black*) and arteries (*white*). A2 to F2 shows time-series analysis of retinal vessel intensity variation (pulsation) over three cardiac cycles. Harmonic analysis yields pulse amplitude heat maps (scale 0–40 arbitrary units) illustrating reduced pulse amplitude from A3 to F3 with more distinct venous occlusion. Distribution of pulse log amplitude versus distance from vessel origin shows flattening with more distinct venous occlusion in arteries (A4–F4) and veins (A5–F5).

The ophthalmodynamometer was calibrated by horizontal placement of the device on a flat surface before use. A range of ODF was applied to the eye from 0 to 50. Retinal video of the optic nerve and peripapillary region was simultaneously captured (Canon 5D Mark III, Japan), mounted on a slit lamp. The video also recorded the audible beeping from the pulse oximeter (Nellcor N65; Covidien, Mansfield, MA) applied to the participants’ index fingers, thereby allowing for synchronization of retinal vascular pulsation with cardiac cycles. This technique was used to measure PPG estimates of blood column pulse amplitude^28^ (Figs. 1A, 1B, Figs. 2A1–F1).

### Image Analysis

**Figure 3.**
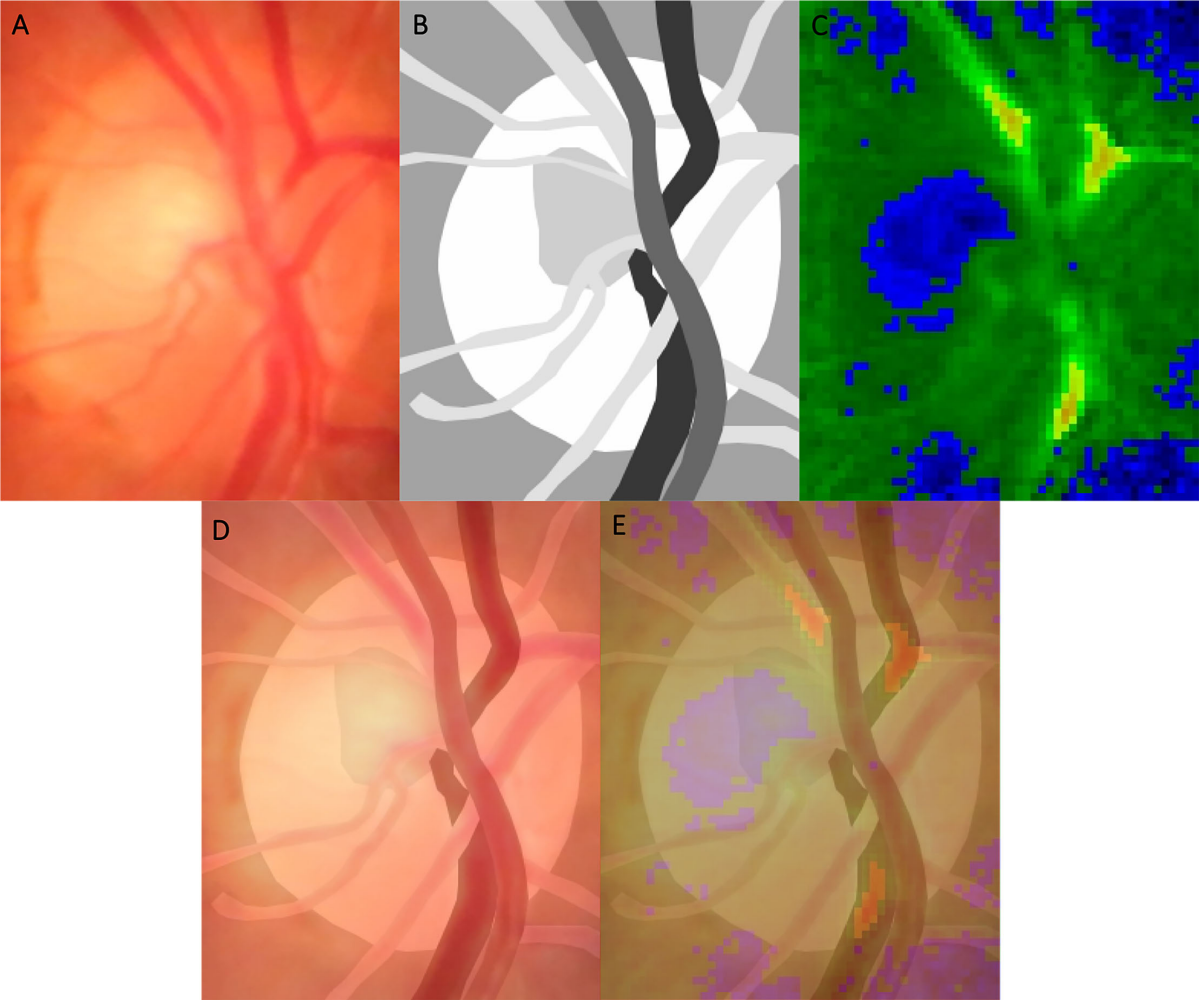
Composite images showing the original video frame (**A**), segmentation of vessels (*dark grey* for artery, *black* for vein, and *light grey* for other vessels not investigated), optic nerve head, cup, and background (**B**), as well as pulse amplitude heat map (**C**). Note that only data from the segmented arteries and veins are analyzed. (**D**) Shows a composite image of the original video frame and segmentation image. (**E**) Shows another composite image of **D** with an overlying pulse amplitude heatmap.

Video frames within three cardiac cycles guided by pulse oximetry audio signals (see Figs. 2A2–F2) were trimmed at different ODFs. The video frames were then exported to Adobe Photoshop CS6 for alignment. Segmentation of artery, vein, optic disc center, and background on images were performed and verified by two observers, Y.J. Khoo and W.H. Morgan (Fig. 3B). The optic disc center served as the polar coordinate center for determining the distance to each locus along the vessels for pulse amplitude and attenuation analysis (see Fig. 1B).

### Statistical Analysis

Statistical analysis was performed using the R package.^30^ Three cardiac cycle time series of aligned green color channels were created for each ODF. Green channel has been found to correspond most strongly to hemoglobin light absorption compared to red and blue color channels, as hemoglobin absorbs green light better than red or blue. Therefore, it has been used to quantify hemoglobin in blood samples.^31^^–^^33^ In this study, green channel densitometry has been used to estimate hemoglobin density at each video frame to detect vessel blood column amplitude change.

Harmonic analysis was performed at each 30 × 30 micron locus (equivalent to a 5 × 5 pixel cluster) within 0.25 to 1 mm from the disc center. This is because we have previously found greatest signal-to-noise ratio in data limited to 0.8 to 1.0 mm from the optic disc center.^34^ We also removed data from 0 to 0.25 mm to eliminate the small rising phase of pulsation and enabled the analysis of the attenuation part of the pulse distribution along the vessel. Peak sensitivity and specificity for pulsation detection was found when pulse amplitude was 5 arbitrary units (95% confidence interval 4.3 to 6.0) or more.^35^ A harmonic regression model consisted of a non-periodic component fitted to a linear spline, a periodic component comprised of the first two harmonics of a trigonometric Fourier series, and a first-order autoregressive error component. A full description of the technique is described in our previous papers.^25^^,^^36^

We applied logarithmic (log) transformation to the harmonic regression wave amplitude to normalize our data, and therefore log unit (log u) was used as our unit of measure for amplitude.^36^ Quantile-quantile plots (q-q plots) were performed to check on the normality of distribution for both arterial and venous amplitudes.

Heat maps of pulse amplitudes were constructed for initial review (Figs. 2A3–F3, Figs. 3C, 3E). The relationship between explanatory variables (study group, age, sex, induced IOP, distance from optic disc center, and the presence of POAG) and response variable (mean, maximum log pulse amplitude, and amplitude slope) was analyzed using linear mixed-effects modeling. The rate of change of amplitude over distance was calculated and termed amplitude slope. The amplitude slopes were calculated by performing linear regression analysis on each vein or artery dataset (log amplitude versus distance) at different induced IOP (ranges from 6–55.5 mm Hg with a mean of 35.4 ± 14.0 mm Hg). Mean and maximum pulse amplitudes were calculated from the same datasets. We included random factors to account for multiple measurements from the same eye at various IOPs and laterality “right/left.” We separated the superior and inferior hemivessel data (as affected and unaffected hemivessels) of HVO eyes for analysis.

We divided the study cohort into six groups, including the normal control group (see Fig. 2, Table):

(1) Normal eyes (N1 N1)
(2) Unaffected eyes in CRVO participants (N1 CRVO)
(3) Unaffected eyes in HVO participants (N1 HVO)
(4) Unaffected hemivessels in HVO eyes (N2 HVO)
(5) Affected hemivessels in HVO eyes (HVO HVO)
(6) CRVO eyes (CRVO CRVO)

**Table.** Number of Eyes and Mean Age in each Study Group, With and Without POAG

Study Group	Eyes With POAG	Eyes Without POAG	Number of Eyes Examined	Mean Age (95% CI)
N1 N1	0	69	69	59.52 (54.93–64.11)
N1 CRVO	4	0	4	57.25 (40.79–73.71)
N1 HVO	5	0	5	73.20 (68.00–78.40)
N2 HVO, HVO HVO	7	1	8	74.38 (70.30–78.45)
CRVO CRVO	8	13	21	70.24 (64.45–76.03)

We also subdivided all groups into glaucomatous and non-glaucomatous (see the Table).

Bonferroni correction of 0.05 ÷ 5 = 0.01 (*P* value < 0.01) was calculated to account for the 5 comparisons performed with each diagnostic group.

## Results

One hundred seven eyes of 70 participants (43 men and 27 women) with a mean age of 64 ± 18 years were examined. Of these, 69 were normal eyes, 21 were CRVO eyes, and 8 were HVO eyes (including 8 unaffected hemiretinal vessels from the same eyes, N2 HVO; see the Table). Eight eyes were excluded from the study due to excessive movement during ophthalmodynamometry, degrading the image quality. A total of 403,636 measurements from the 107 eyes were taken, ranging from 722 to 12,194 from each eye. The distribution of distances from which measurements were taken was not significantly different between diagnostic subgroups (minimum *P* = 0.0394).

The venous pulsation of the normal study group showed a slight increase in amplitude of 0.080 log u/year (*P* = 0.0080) with age but not with other study groups. The arterial pulse amplitude association on the other hand did not reach statistical significance with age among any participant groups (minimum *P* = 0.04 for the normal group).

### Venous Analysis

#### Mean Venous Log Harmonic Amplitude

**Figure 4.**
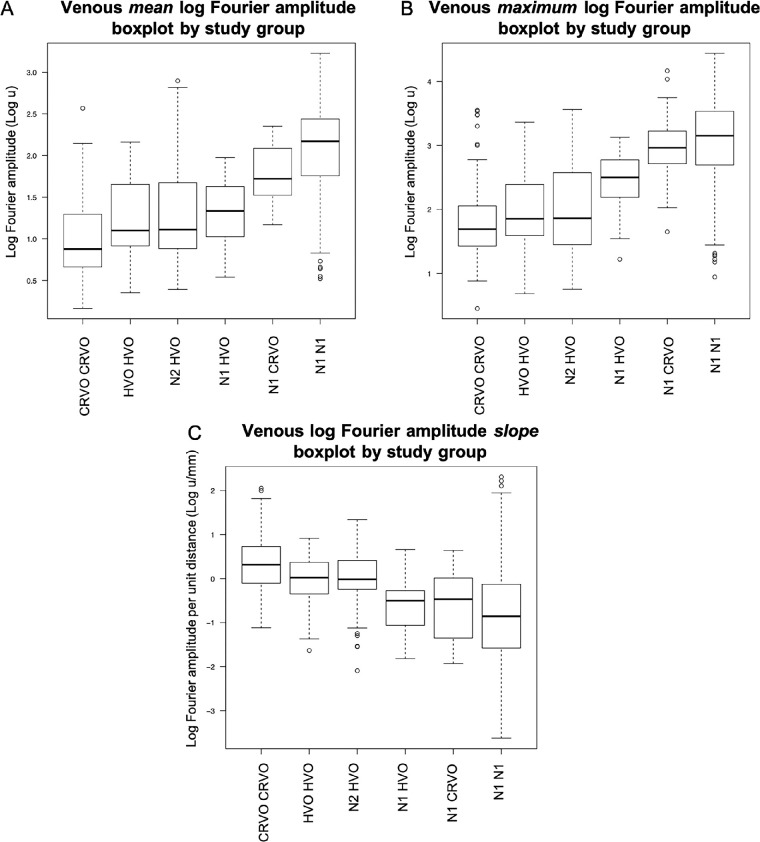
Box plots of the venous mean (**A**) and maximum (**B**) log harmonic amplitudes in each diagnostic group. Log amplitudes decrease with more distinct venous occlusion from normal to CRVO eyes. Venous log harmonic amplitudes per unit distance (**C**) along vessels in each study group show increasing slopes with more distinct venous occlusion from normal to CRVO eyes.

**Figure 5.**
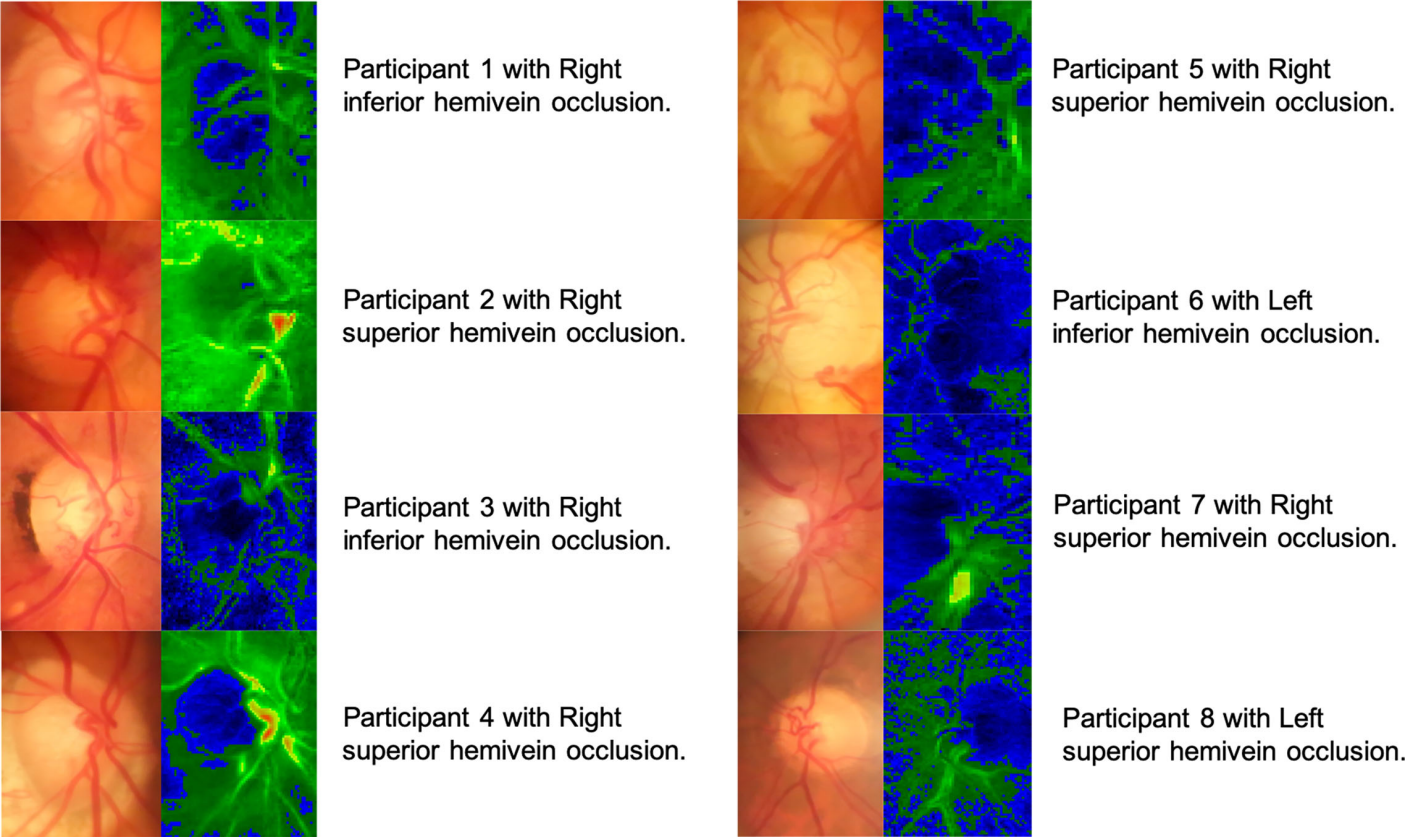
Composite image showing qualitative comparison of heat maps between N2 HVO and HVO HVO. Note that there is a visible difference between the superior and inferior vessel pulsation amplitude for most participants except for participants 4 and 6, although this did not reach statistical significance.

As expected, the normal group, N1 N1, demonstrated the highest mean venous pulse log amplitude of all study groups (2.08 ± 0.48 log u). In comparison, CRVO eyes had the lowest mean log amplitude (0.99 ± 0.45 log u, *P* < 0.0001), followed by HVO hemiveins (1.23 ± 0.46 log u, *P* = 0.0002) and the unaffected hemiveins in HVO eyes (N2 HVO; 1.3 ± 0.59 log u, *P* = 0.0005). This trend of reducing venous mean log pulse amplitude with more distinct venous occlusion (from N1 N1 to CRVO) is shown in Fig. 4A. There was no statistically significant difference in venous mean log amplitude between eyes with CRVO and HVO (*P* = 0.48), as well as between HVO and N2 HVO hemiveins (*P* = 0.06; Fig. 5).

Interestingly, the unaffected eyes of venous occlusion participants, N1 CRVO (1.76 ± 0.34 log u, *P* = 0.52) and N1 HVO (1.33 ± 0.37, *P* = 0.0101) demonstrated a lower venous mean log amplitude compared to N1 N1 (2.08 ± 0.48 log u) in the absence of clinical evidence of occlusive vasculopathy. However, this subanalysis failed to achieve statistical significance. N1 CRVO eyes had a significantly higher mean log amplitude compared to CRVO eyes (*P* = 0.0004) and HVO hemiveins (*P* = 0.0062), although comparable to the unaffected hemiveins of HVO eyes (N2 HVO, *P* = 0.0130). The contralateral unaffected eyes of HVO (N1 HVO) were higher though not statistically significant in mean log amplitude compared to CRVO CRVO (*P* = 0.13), HVO HVO (*P* = 0.23), and N2 HVO (*P* = 0.43) eyes.

#### Maximum Venous Log Harmonic Amplitude

The maximum log pulse amplitude analysis showed comparable results compared to mean log amplitude, with three exceptions (see Fig. 4B):

(1) The N2 HVO group had a significantly higher maximum (*P* = 0.0024), although not significantly different from HVO HVO in mean log amplitude (*P* = 0.0638).
(2) The maximum log amplitude of N2 HVO was significantly lower than N1 CRVO (*P* = 0.0074). However, the mean log amplitude of N2 HVO was not statistically different compared to N1 CRVO (*P* = 0.0130).
(3) The maximum log amplitude of the unaffected eyes of subjects with occlusive retinal vasculopathy (N1 CRVO and N1 HVO) were significantly higher than their diseased counterparts (N1 CRVO versus CRVO CRVO, *P* = 0.0007; and N1 HVO versus HVO HVO, *P* = 0.0055). With mean log amplitude, N1 HVO was not significantly different from HVO HVO (*P* = 0.23).

There were no significant relationships found between mean or maximum log venous pulsation amplitudes with age (*P* > 0.46), sex (*P* > 0.32), and POAG (*P* > 0.55) for any study group.

#### Venous Log Harmonic Amplitude Slope

The slopes of venous log harmonic amplitude per unit distance along vessels in each study group increased with more distinct venous occlusion (from normal to CRVO eyes; Fig. 4C). N1 N1 had the greatest log amplitude attenuation (negative slope) along their veins (−0.80 ± 1.03 log u/mm, 95% confidence interval [CI] = 0.56, −1.04), with only CRVO demonstrating statistical significance compared to N1 N1. Interestingly, CRVO was the opposite of N1 N1 and had a positive log amplitude change (0.35 ± 0.6 log u/mm, *P* < 0.0001, 95% CI, 0.61, 0.09) rather than attenuation. HVO and N2 HVO had flatter slopes compared to N1 N1 (HVO 0.000 ± 0.51 log u/mm, *P* = 0.0391; and N2 HVO -0.005 ± 0.64 log u/mm, *P* = 0.0202). N1 CRVO (−0.54 ± 0.76 log u/mm, *P* > 0.1) and N1 HVO (−0.63 ± 0.55 log u/mm, *P* > 0.04) had greater attenuation of log amplitudes than eyes affected by venous occlusion (CRVO, HVO, and N2 HVO), although they were not statistically significant (Fig. 4C).

### Arterial Analysis

#### Arterial Mean and Maximum Log Harmonic Amplitude

**Figure 6.**
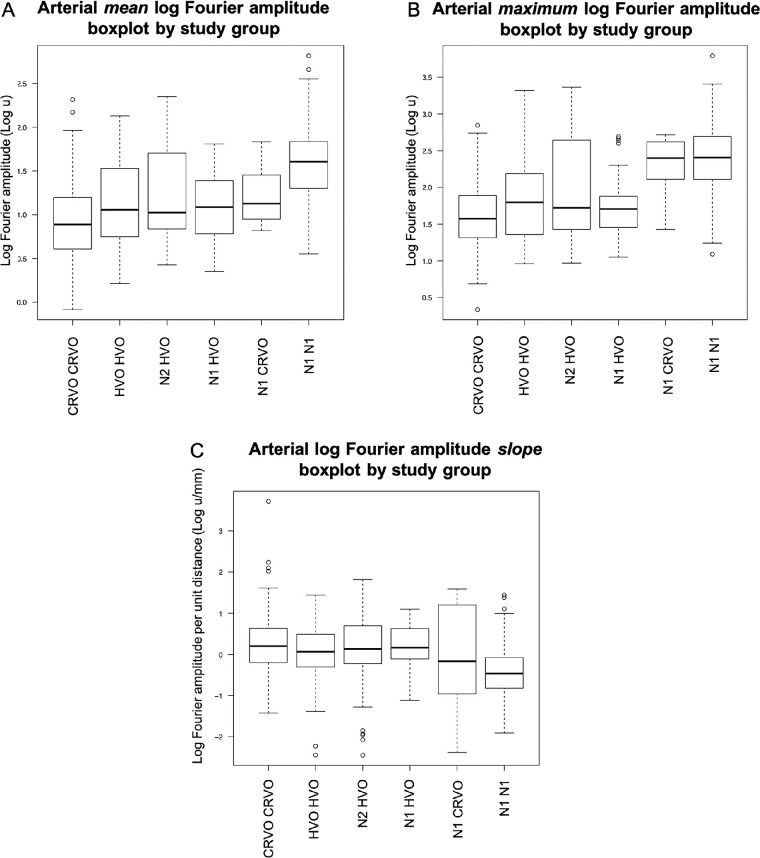
Box plots of the arterial mean (**A**) and maximum (**B**) log harmonic amplitudes in each diagnostic group. Log amplitudes tend to decrease with more distinct venous occlusion from normal to CRVO eyes. Arterial log harmonic amplitudes per unit distance (**C**) along vessels in each study group show increasing slopes with more distinct venous occlusion from normal to CRVO eyes.

The arterial mean and maximum log harmonic amplitudes showed similar trends to their venous counterparts (Figs. 6A, 6B versus Figs. 4A, 4B). The normal eyes, N1 N1 had the highest mean and maximum log amplitudes (1.56 ± 0.39 and 2.38 ± 0.43 log u, respectively). CRVO eyes had the lowest mean and maximum log amplitudes (0.91 ± 0.44 and 1.60 ± 0.44 log u, respectively). Surprisingly, although not clinically involved, arteries of eyes affected by venous occlusion had significantly lower mean and maximum log amplitudes compared to N1 N1 (mean: N2 HVO 1.23 ± 0.51 log u, *P* = 0.0074, HVO HVO 1.15 ± 0.49 log u, *P* = 0.0047, CRVO CRVO 0.91 ± 0.44, *P* < 0.0001; maximum: N2 HVO 1.94 ± 0.63 log u, *P* = 0.0097, HVO HVO 1.84 ± 0.03 log u, *P* = 0.0029, CRVO CRVO 1.6 ± 0.44 log u, *P* < 0.0001). N1 HVO and N1 CRVO had lower mean and maximum log amplitudes than N1 N1, but they did not reach statistical significance (N1 HVO mean 1.09 ± 0.37 log u; max 1.73 ± 0.4 log u, *P* > 0.0167; N1 CRVO mean 1.23 ± 0.3 log u; max 2.31 ± 0.35 log u, *P* > 0.20).

There were no significant relationships between arterial mean or maximum log pulsation amplitudes with age (*P* > 0.58), sex (*P* > 0.60), and POAG (*P* > 0.20) for any study group.

#### Arterial Log Harmonic Amplitude Slope

The slopes of arterial log harmonic amplitude per unit distance along vessels in each study group increased from normal to CRVO eyes, similar to the venous slopes (Fig. 6C versus Fig. 4C). Normal eyes had the greatest log amplitude attenuation (negative slope) along their arteries (−0.44 ± 0.54 log u/mm, 95% CI = −0.31 to −0.57). CRVO was the opposite of N1 N1 and had a positive slope (0.26 ± 0.7 log u/mm, *P* = 0.0036, 95% CI = 0.56 to −0.04) rather than attenuation.

### Arterial Versus Venous Analysis Within Study Groups

An analysis comparing the arterial and venous pulsation amplitude within groups showed that the arterial mean log harmonic amplitudes were lower than veins in all groups (*P* < 0.0028; Figs. 4A, 6A) As for the maximum log harmonic amplitude, the arteries were found to be lower than veins in all groups (*P* < 0.0001) except for HVO HVO (*P* = 0.0114; Figs. 4B, 6B).

**Figure 7.**
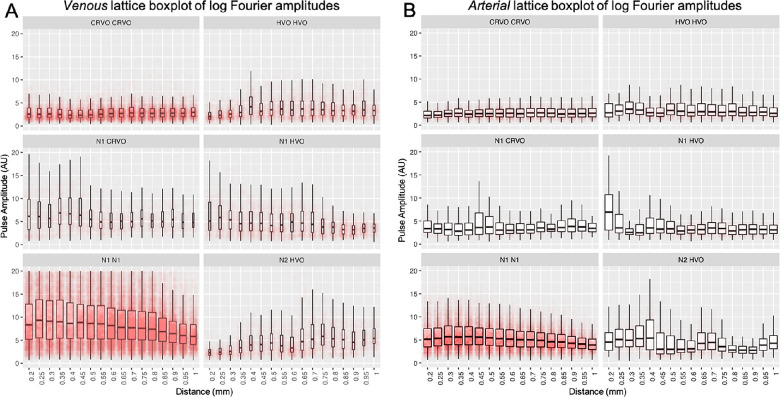
Lattice boxplots of log harmonic amplitudes vs distance along the veins (**A**) and arteries (**B**) at binned intervals of 0.05 mm, starting at 0.2 mm from the disc center. These demonstrate the distribution of log pulse amplitudes at each of the 0.05 mm points along the vessels.

Arterial amplitude slope analysis ranged from -0.44 to 0.26 log u/mm, whereas venous analysis ranged from −0.8 to 0.35 log u/mm from N1 N1 to CRVO (Figs. 4C, 6C, 7A, 7B). Venous pulse amplitude distributions were significantly more attenuated than the arteries among the N1 N1 (*P* < 0.0001) and N1 HVO (*P* < 0.0001) participants. The veins of CRVO participants had a more positive slope than the arteries (*P* = 0.0005). N1 CRVO (*P* = 0.10), N2 HVO (*P* = 0.73), and HVO HVO (*P* = 0.02) did not reach statistical difference between arteries and veins.

## Discussion

This work demonstrates reduced retinal arterial and venous pulse amplitudes in eyes affected by central forms of venous occlusion (CRVO and HVO), including the unaffected hemivessels of HVO eyes. These observations in retinal veins are consistent with elevated venous pulsation pressure observed in previous work.^24^^,^^27^^,^^37^ In addition, we also demonstrate that arterial pulse amplitudes are reduced in central forms of venous occlusion. To the best of our knowledge, this is the first work to include HVO and the unaffected hemivessels of the HVO eyes quantified in the Fourier domain.

Arterial and venous groups affected by venous occlusion (CRVO, HVO, and N2 HVO) had significantly reduced mean and maximum amplitudes compared to N1 N1. The arterial mean and maximum amplitudes showed reduced amplitudes overall compared to veins. In a normal situation, the transmural pressure in the central retinal vein is close to zero, allowing significant volume change per unit change in transmural pressure. Therefore veins exhibit significant pulse amplitude changes with the cardiac cycle under normal circumstances.^21^^,^^38^ With occlusion, veins become engorged, the transmural pressure difference increases, the distensibility or volume change per unit change plateaus, and the pulsation amplitude decreases.^21^ Retinal arteries have a higher transmural pressure^21^ resulting in lower distensibility than veins. This is exacerbated in venous occlusion due to increased vascular pressure upstream along the vascular tree. In addition, the arterial wall tends to be stiffer than veins with lower compliance.^38^

Mozaffarieh et al.^27^ found a significant difference in the venous pulsation of the unaffected eyes of CRVO participants compared to their control group. In our study, whereas N1 CRVO and N1 HVO tended to have lower mean and maximum amplitudes in both arteries and veins compared to N1 N1, this did not reach statistical significance. Our findings could have been confounded by the low number of eyes in both groups: four in the N1 CRVO group and five in the N1 HVO group. (Mozaffarieh et al. had 31 participants with CRVO and N1 CRVO eyes). Although the unaffected eyes were not statistically different, the unaffected hemiveins and arteries of HVO eyes (N2 HVO) were found to have significantly lower mean and maximum amplitudes than N1 N1. The reduced pulse amplitudes suggest elevation of transmural pressure in supposedly unaffected vessels in HVO eyes. This may indicate a hemodynamic effect of HVO on the “unaffected” part of the vascular tree. We are uncertain but suspect that anastomosis formation toward the central vein may elevate overall retinal venous pressure. HVO tends to have a greater rate of improvement with treatment in visual outcomes than either CRVO or branch vein occlusion, possibly due to the enhanced ability to develop collateral vasculature within the anterior part of the optic nerve, which may have an associated effect on the pulse amplitudes.^39^

Normal healthy eyes had the greatest reduction in pulse amplitude with distance from the disc center (attenuation) in both arteries and veins. Other study groups, including CRVO, HVO, N2 HVO, N1 CRVO, and N1 HVO, showed flattening of this relationship, although only CRVO was statistically significant when compared to N1 N1 for arteries and veins. The amplitudes of CRVO arteries and veins tended to increase (positive amplitude slopes) rather than attenuate. However, only the slope of venous pulsation amplitude was significantly different from zero, whereas the slope of arterial pulse amplitude was not. With vessel engorgement and dilation, transmural pressure increases, compliance reduces and hence vessel walls become stiffer. Stiffer vessels have reduced pulse amplitudes but transmit pulsation with reduced energy loss, reducing pulse wave attenuation. This may explain the flatter pulse amplitude distribution in the arteries but not the increased amplitudes seen in the veins. Another possible cause of increased amplitudes with distance seen in veins may be wave reflection from narrower regions and tributary junctions upstream in the venous part of the circulation.^40^

Glaucoma has been shown to be a significant risk factor for retinal venous occlusion. However, the presence of POAG did not influence the mean or maximum amplitudes for both venous (*P* > 0.55) and arterial analysis (*P* > 0.20) for any study groups in this study. One limitation of the glaucoma analysis is that the N1 N1 group does not have participants with glaucoma, whereas all the unaffected eyes of patients with venous occlusion had POAG.

Other limitations of this study include the low number of eyes in each subgroup, especially in the N1 CRVO and N1 HVO groups, which may account for their statistical nonsignificance when compared to diseased and normal eyes. Another limitation is the younger age group of the normal and N1 CRVO eyes compared to the diseased groups (CRVO, HVO, and N2 HVO; see the Table). As such, we included age as an explanatory variable to account for the variation in age.

Photoplethysmographic measures of vessel pulse amplitude changes with central retinal venous occlusion, consistent with the known effects upon transmural pressure and changes in compliance. Such measurements may be useful in evaluating the severity, progression, and response to therapy in eyes with venous occlusion.
